# The Feasibility of Developing a Construction Material From Basaltic Quarry Waste and Recycled High-Density Polyethylene

**DOI:** 10.1155/tswj/5519409

**Published:** 2025-02-24

**Authors:** Johnson Ngugi, George O. Rading, Thomas O. Mbuya

**Affiliations:** Department of Mechanical and Manufacturing Engineering, University of Nairobi, Nairobi, Kenya

**Keywords:** basaltic quarry waste, density, FTIR, mechanical properties, recycled HDPE, thermal stability, water absorption

## Abstract

Quarry waste is a fine rock aggregate produced as a by-product of the rock-crushing process in quarries which is environmentally hazardous when poorly disposed. This paper presents the results of a study carried out to explore the feasibility of recycling basaltic quarry waste (BQW) with waste high-density polyethylene (HDPE) into sustainable construction materials. Recycled HDPE/BQW formulations were melt-mixed in a single-screw extruder and then transfer-moulded into experimental samples. No significant chemical transformations were detected by Fourier transform infrared spectroscopy. Thermogravimetric analyses showed an improvement in the thermal stability of HDPE with the addition of BQW. Scanning electron microscopy imaging revealed generally poor adhesion between the two phases. Both tensile and impact strength initially increased but decreased at higher filler loading. However, stiffness, compressive strength, compressive modulus, density, and hardness improved with filler content for all particle sizes. The increase in water absorption with increasing filler content was not significant. This study demonstrates that BQW is a suitable filler for HDPE and the resulting material may be used to make roofing tiles and paving blocks.

## 1. Introduction

When different types of igneous rocks are extracted, crushed, and sorted into various grades of construction aggregates in quarries, the fine aggregate that passes through the 4.75-mm sieve is called quarry waste (QW) (or quarry dust) [1, 2]. The quarry waste generated during this process is substantial and can account for up to 25% of the final crushed aggregates [3]. Since QW has traditionally had little value beyond usage in road construction and for making building blocks [4], it is considered a waste material and large quantities of QW are disposed in landfills every year [1]. Improperly disposed QW is hazardous to the environment and contaminates water and air, unclogs soil, and degrades land [5].

Due to rising urbanization in developing countries which is rapidly depleting traditional construction materials like sand [1], QW has, in the recent past, attracted attention alongside other wastes such as fly ash [6], copper slag [7], marble waste [8], and glass waste [9] as potential inclusions in concrete. As a result, Balamurugan and Perumal [2]; Aishwaryalakshmi et al. [3]; Rahim et al. [10]; Prakash and Rao [11]; and Meisuh, Kankam, and Buabin [12] have shown that QW can replace up to 50% of sand in concrete without any significant decline in concrete's mechanical properties. Sundaralingam et al. [13] also recently demonstrated that QW can replace sand in cement–sand masonry blocks. Whereas these developments will enhance the use of QW, there is a need to develop multiple recycling channels if the massive volumes of QW produced annually are to be sufficiently exploited.

A promising alternative application for QW is as a filler for recycled plastics. The negative environmental effects of conventional plastic waste management methods such as landfilling and incineration are well documented [14]. There is, therefore, a global shift towards a circular plastic economy which encourages the reuse and recycling of plastics into new materials in ways that will both meet consumption demands and conserve the environment [15]. While a large volume of data on the recycling of plastic waste with different fillers was identified by the authors in literature, this research has not translated into sufficient viable waste plastic recycling channels. This is demonstrated by a recent report published by the Organisation for Economic Cooperation and Development (OECD) which shows that only 9% of all waste plastics generated globally are currently recycled. About 22% are mismanaged and the rest are either incinerated or landfilled [16]. There is, therefore, a need to develop alternative waste plastic–based products with a high market potential to provide the much-needed incentive for recycling. QW is cheap and widely available and thus a suitable material for recycling alongside plastic waste into low-cost products.

The material developed in this study was specifically targeted for producing low-cost construction products such as roofing tiles and paving blocks to substitute similar products made from conventional materials. High-density polyethylene (HDPE) was selected because it has many attractive properties [1] which do not significantly degrade during service and is, therefore, a good candidate for recycling. Whereas a significant body of research exists in literature where postconsumer waste HDPE was recycled with fibrous fillers such as rice husks [17, 18], hemp [19, 20], sisal [21], and particulate fillers such as wood particles [22, 23], nanoalumina particles [24], carbon black waste [25], mica [26], and fly ash [27] into composite materials targeted for diverse applications, no information on the properties of QW with recycled HDPE was traced. In all three studies where QW was explored as a filler for HDPE [28–30], virgin HDPE, which is costlier than recycled HDPE, was used. Furthermore, the properties of the composite materials developed in these studies were not sufficiently characterised. For instance, the QW used by Xuen, Hoe, and Munusamy [30] was not identified. Further, both Xuen, Hoe, and Munusamy [30] and Shagwira et al. [28] reported only two mechanical properties. While de Moura et al. [29] reported additional properties, the particle size ranges selected were wide and overlapping and thus, the effect of particle size on composite properties was not clearly defined. Whereas Shagwira et al. [28] and de Moura et al. [29] used granitic quarry waste (GQW), basaltic quarry waste (BQW) is used in the current study. Narrower particle size ranges are selected, and composite properties not previously reported are investigated.

## 2. Materials and Methods

### 2.1. Materials

**Figure 1:**
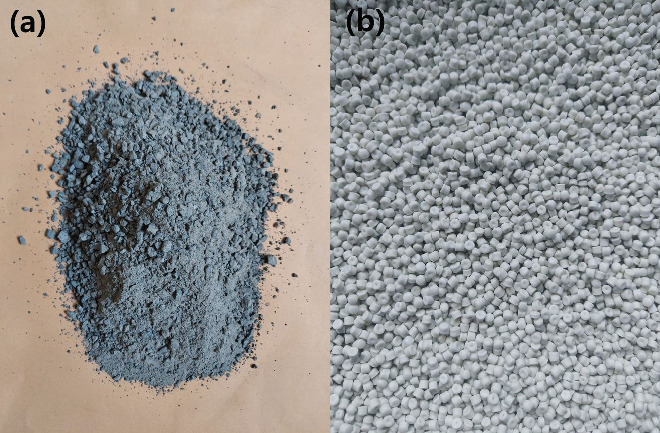
Material used to make HDPE/BQW composites: (a) BQW; (b) recycled HDPE pellets.

The methodology adopted for this study is similar to the one used in a previous study by the authors in [31] where QW was used as a filler for recycled polypropylene (PP). The same BQW acquired from Silver Stone Quarry Limited in Machakos County, Kenya, whose chemical composition is presented in Table 1, was used. Recycled HDPE pellets of about 3 mm in diameter and 3 mm in length were obtained from Mr Green Africa Limited in Nairobi, Kenya. The pellets had a melting temperature of 131°C and a melt flow rate of 1.5 g/10 min (at 190°C). The BQW and recycled HDPE pellets used are shown in Figures 1(a) and 1(b), respectively.

### 2.2. Preparation of Recycled HDPE/BQW Composites

The BQW was sorted using sieves into four narrow particle size ranges to provide a clear picture of the effect of particle size on composite properties. The particle sizes used were 300–310, 150–160, 50–60, and <50 *μ*m, the choice of which was determined by the availability of sieves. Water-soluble particles and particulate suspensions were removed by washing each BQW sample with distilled water until the water was clear. The washed BQW samples were then dried in an LTE OP60-U/M oven at 106°C for 24 h. The HDPE pellets were also dried in the oven for 24 h at 50°C to remove moisture.

Four initial recycled HDPE/BQW formulations of 100/0, 90/10, 80/20, and 70/30 wt% were selected to study the variation of mechanical properties with increasing filler content. Subsequently, five additional formulations between 100/0 and 90/10 wt% were prepared to locate the compositions at which the composites exhibited the optimum tensile strength. All the formulations prepared are presented in Table 2. The HDPE/BQW formulations were manually mixed and then melt-blended at 190°C in a locally fabricated single-screw extruder with a length-to-diameter ratio of 35:1 and a screw diameter of 25 mm running at a speed of 56 rpm. The extrudate was collected and transfer-moulded using a hydraulic press into a mild steel mould cavity machined to form experimental samples. The moulding pressure used was 200 bar, and moulding was done at room temperature (23°C). The moulding pressure was applied for at least 5 min to allow the composites to solidify. This process was repeated to produce a minimum of five samples for each formulation.

### 2.3. Composite Characterization

#### 2.3.1. Fourier Transform Infrared Spectroscopy (FTIR)

The FTIR analyses of BQW and unfilled HDPE were carried out using a Bruker Alpha FTIR spectrometer. Thin representative composite samples of particle size range < 50*  μ*m were also tested to determine whether any chemical changes occurred or new interfaces were formed between HDPE and BQW during extrusion and moulding. The FTIR spectra were recorded in reflectance mode after 15 scans in the range 400–4000 cm^−1^ at a resolution of 2 cm^−1^. Before spectrum acquisition for each sample, a background spectrum was collected and used to subtract any unwanted residual peaks from the sample spectrum.

#### 2.3.2. Thermogravimetric Analysis (TGA)

TGAs of unfilled HDPE and composite samples of particle size range < 50*  μ*m were conducted using a Shimadzu TGA-50 thermogravimetric analyser. Samples were heated in alumina crucibles from room temperature (24°C) to 600°C at a heating rate of 10°C/min under a cloud of nitrogen flowing at 50 mL/min. To eliminate the buoyancy effects of the surrounding gas, a blank experiment was run and subsequently subtracted from the sample curve.

#### 2.3.3. Mechanical Characterization

##### 2.3.3.1. Tensile Properties

The moulded tensile test samples were dog bone shaped conforming to the Type B specimen specified by British Standard (BS) European Standard (EN) ISO 3167:2014 with an overall length of 150 mm, a gauge length of 50 mm, and a thickness of 4 mm. Samples were tested at a speed of 5 mm/min using a retrofitted Torsee material testing machine equipped with a 10-kN load cell.

##### 2.3.3.2. Compression Properties

Compression test specimens were prepared according to ISO 604:2002 for moulded plastics with the Type B specification (length = 10 mm, width = 10 mm, and thickness = 4 mm). Tests were conducted at a test speed of 1 mm/min using a Torsee material testing machine equipped with a 10-kN load cell.

##### 2.3.3.3. Impact Properties

The double-notched test sample (80 mm long, 10 mm wide, and 4 mm thick) of notch Type B specified by BS EN 179-1:2010 was selected to study the impact properties of the composites. A 62-mm span length was used, and Charpy's impact tests were conducted using a pendulum impact tester (Gunt WP 400) equipped with a 25-Nm hammer. Impact data was recorded using a WP 400/410.20 data acquisition system.

##### 2.3.3.4. Hardness Properties

Hardness tests of both the unfilled and filled plastics were conducted according to the requirements of ASTM D2240. The test specimens had a diameter of 50.8 mm and a thickness of 6 mm, and Shore *D* Durometer test equipment was used. The specimens were placed horizontally on a flat metal surface, and at least 10 hardness values were measured at various points about 12 mm from the edge of the sample, ensuring the durometer was perpendicular to the metallic surface at all times.

#### 2.3.4. Water Absorption

The water absorption test specimens were disc-shaped having a thickness of 3.2 mm and a diameter of 50.8 mm as specified by ASTM D50-98:2018. Before the test, the test specimens were conditioned in an oven for 24 h at 50°C before they were weighed using a Citizen electronic balance (model CX 265) to the nearest 0.00001 g and placed in a desiccator. Immersion was conducted in distilled water for 24 h at 24°C before the samples were wiped with a dry cloth and weighed again to the nearest 0.00001 g. The samples were reimmersed and reweighed after 7 days. This process was repeated every 14 days until the percentage increase in weight was lower than 1% of the total increase in weight as recommended by ASTM D50-98:2018 to achieve saturation. The last sample achieved saturation after 166 days.

The increase in weight was calculated using Equation (1).

(1) $$\begin{array}{c} \text{Water absorption }\left(\%\right)=\frac{\text{weight after immersion}-\text{weight before immersion}}{\text{weight before immersion}}\times 100 \end{array}$$

#### 2.3.5. Density

The water absorption specimens whose specifications are described above were used for this test. Their masses were measured using the Citizen electronic balance, and the volume of water displaced when each specimen was immersed in water in a graduated measuring cylinder was recorded. The densities of the specimens were calculated using Equation (2).

(2) $$\begin{array}{c} \text{Density }\left(\text{kg}/{\text{m}}^{3}\right)=\frac{\text{condtioned weight of specimen}}{\text{volume of displaced water}} \end{array}$$

#### 2.3.6. Scanning Electron Microscopy (SEM) Characterization

Before SEM analysis, 45-nm-thick platinum coatings were applied on selected Charpy's impact and tensile fracture surfaces using a turbo-pumped Q 150T sputter coater. The specimens were imaged using a field emission scanning electron microscopy (FESEM) (S-4000, Hitachi, Tokyo, Japan) at an accelerating voltage of 20 kV.

## 3. Results and Discussion

### 3.1. FTIR Spectroscopy

**Figure 2:**
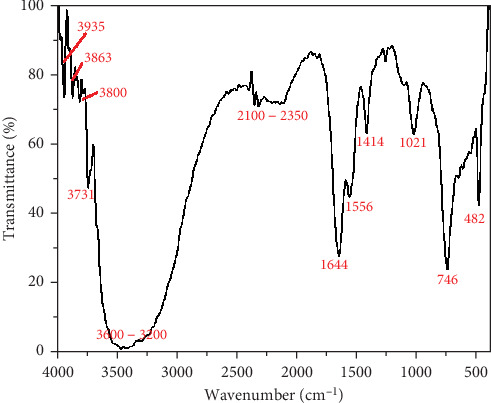
FTIR spectrum of BQW.

**Figure 3:**
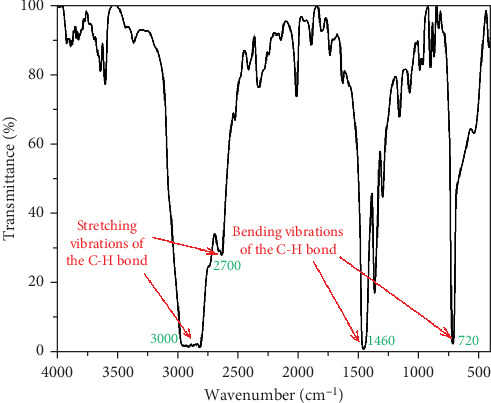
The FTIR spectrum of unfilled HDPE.

**Figure 4:**
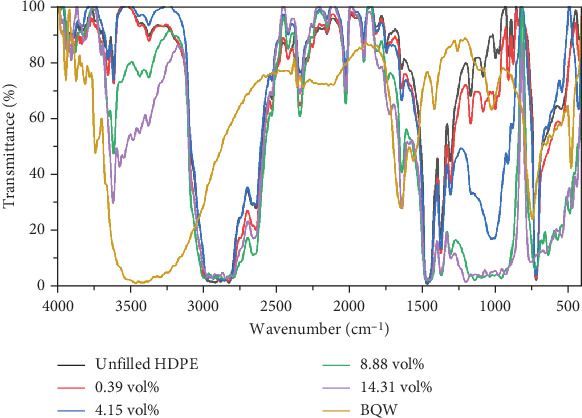
A comparison of the FTIR spectra of BQW, recycled HDPE, and HDPE/BQW composites.

The FTIR spectra of BQW and unfilled HDPE are shown in Figures 2 and 3, respectively. The FTIR spectra of BQW and HDPE plotted alongside those of selected composites are presented in Figure 4.  Table 3 summarises the functional groups detected in BQW. The series of small absorption bands observed in Figure 2 at wavelengths 3935 cm^−1^, 3863 cm^−1^, 3800 cm^−1^, and a more prominent one at 3731 cm^−1^ are typical of the metal hydroxide (M-OH) stretching vibrations of biotite [32, 33]. The adjacent high intensity and broad absorption band between 3600 and 3200 cm^−1^ is characteristic of the stretching vibrations of hydroxyl groups [34] signifying the hydrophilic nature of BQW. Between 2100 and 2350 cm^−1^, there is a series of low-intensity peaks which may suggest the presence of an organic compound. The sharp peaks with decreasing intensities at 1644, 1556, and 1414 cm^−1^ are likely due to the stretching of the (CO_3_)^−2^ bond and indicate the presence of calcite in BQW [34]. The sharp peaks at 1021, 746, and 482 cm^−1^ are consistent with the presence of the Si-O-Si functional group in quartz which is the principal oxide in Table 1. Specifically, the peaks at 1021 and 746 cm^−1^ are due to asymmetrical stretching vibrations while the one at 482 cm^−1^ is in symmetrical bending mode [33, 34].

The four main peak characteristics of neat HDPE can be seen in the FTIR spectrum of unfilled HDPE in Figure 3. The double peak between 2700 and 3000 cm^−1^ is associated with the stretching vibrations of the C-H bond, while the ones at 1460 and 720 cm^−1^ are attributed to the bending vibrations and bending of the C-H bond, respectively [35]. The extra peaks with low intensity observed at 3610, 2330, 2020, 1630, and 1000 cm^−1^ are not typical of neat HDPE and likely belong to the functional groups introduced by polymer additives.

Comparing the FTIR spectra of BQW and HDPE against those of the filled polymers in Figure 4, there are no new distinct peaks that would typically be associated with covalent modifications between HDPE and BQW. The overall effect of BQW on recycled HDPE was that it increased the peak intensities of the extra peaks (peaks deemed to have been introduced by polymer additives) with increasing filler loading. This may mean that BQW particles multiplied the number of functional groups present in these compounds [36].

### 3.2. TGA

**Figure 5:**
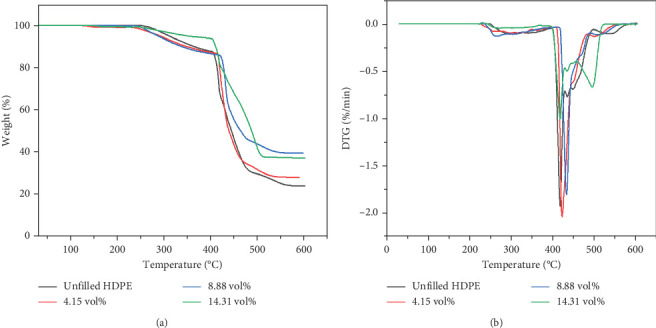
Thermogravimetric analyses of filled and unfilled HDPE: (a) TGA curves; (b) DTG curves.

The previous study [31] showed that BQW had high thermal stability with a minimal mass loss of 1.61% at 600°C. Figures 5(a) and 5(b) illustrate the TGA and differential thermogravimetric analysis (DTG) curves of unfilled and BQW-filled HDPE, respectively, and Table 4 presents the thermogravimetric data. Table 4 shows that unfilled HDPE started degrading at about 246°C. According to Figure 5(a), HDPE decomposed in a three-stage process where a rapid mass loss process (between 403°C and 483°C) was sandwiched between the first (between 252°C and 403°C) and the third (483°C and 565°C) slower processes. This multistage decomposition is confirmed by the DTG curves in Figure 5(b) which shows smaller mass loss peaks on either side of a central pronounced peak. Given that recycled HDPE typically starts to degrade at temperatures above 350°C and in a single-stage process [18, 19, 26], it is highly likely that the material that began to decompose at 246°C was not HDPE but at least one of the unidentified polymer additives that exhibited the extra peaks in the FTIR spectrum of unfilled HDPE in Figure 3. Therefore, HDPE likely started to break down at the onset of the second stage of decomposition at 403°C. In that case, Table 4 shows that the addition of BQW slightly accelerated the thermal degradation of these unidentified polymer additive materials. This was likely caused by the vaporization of volatile compounds in BQW such as phosphorus oxide in addition to the combustible substances that were lost on ignition in Table 1. However, BQW generally enhanced the thermal stability of HDPE in the second and third stages of decomposition as indicated by the higher peak temperatures and higher residual mass in Table 4. This effect increased with increasing filler loading and is consistent with the assertion that inorganic fillers improve the thermal stability of polymers by dissipating heat away from the polymer (since they have a higher thermal conductivity) and shielding the volatile products generated by the decomposing polymer from diffusing out of the composite [37].

### 3.3. SEM Analysis

**Figure 6:**
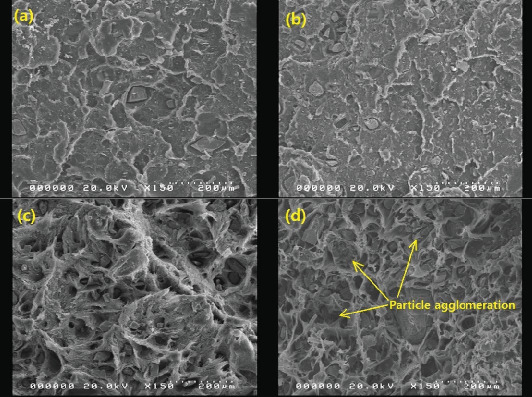
SEM images of impact fracture surfaces of recycled HDPE/BQW composites with (a) 50–60-*μ*m particles at 0.39 vol.% BQW content, (b) 50–60-*μ*m particles at 1.19 vol.% BQW content, (c) 50–60-*μ*m particles at 8.8 vol.% BQW content, and (d) 50–60-*μ*m particles at 14.31 vol.% BQW content.

**Figure 7:**
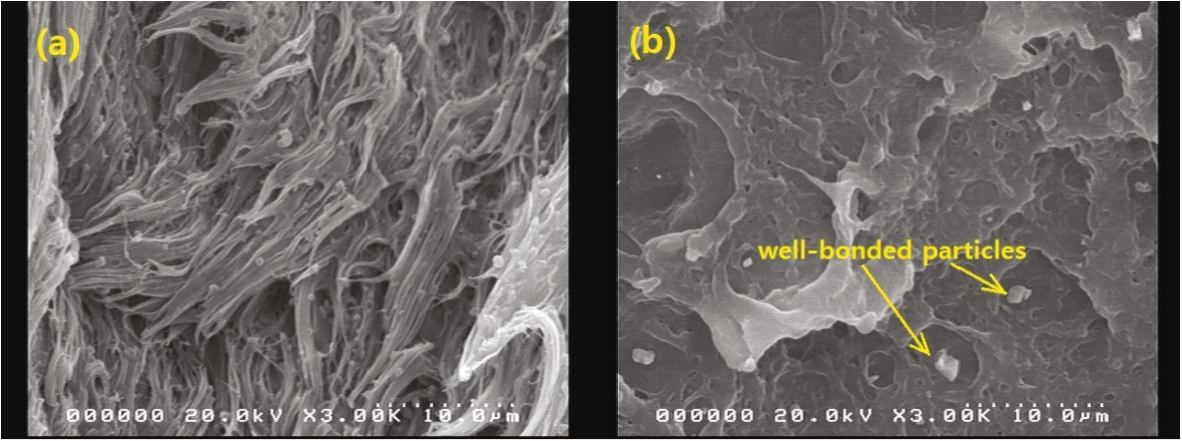
SEM image of recycled HDPE/BQW composite with < 50-*μ*m particles at 0.39 vol.% BQW content: (a) tensile fracture surface; (b) impact fracture surface.

**Figure 8:**
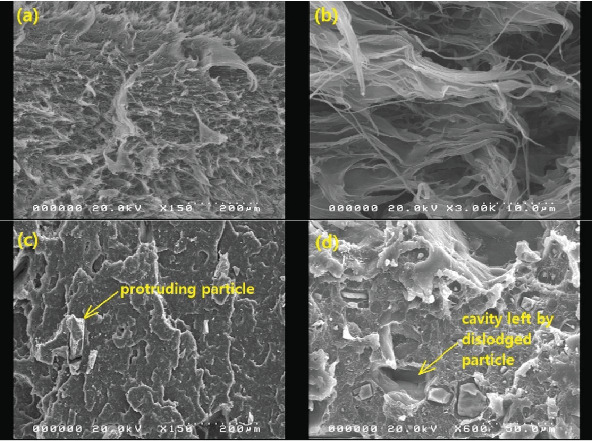
SEM images of impact fracture surfaces of (a) unfilled HDPE at a low magnification, (b) unfilled HDPE at a high magnification, (c) HDPE/BQW composite with 150–160-*μ*m particles at 0.39 vol.% BQW content, and (d) HDPE/BQW composite with <50-*μ*m particles at 0.39 vol.% BQW content.

Selected SEM images of the composites are presented in Figures 6, 7, and 8. It is notable in Figures 6(a) and 6(b) that the distribution of BQW particles in the HDPE matrix was not homogeneous. This implies that the single-screw extruder with a high length-to-diameter ratio employed was not able to sufficiently counteract the low mixing efficiency typically associated with single-screw extruders [38]. The dispersion of filler particles within the matrix plays a key role in determining composite properties. The better the dispersion, the greater the reinforcing effect and the better the mechanical performance [39, 40]. Therefore, the poor dispersion observed in these composites is likely to negatively impact their mechanical performance. The adhesion between the two phases seems to be comparatively better in Figures 6(a) and 6(b) than in Figures 6(c) and 6(d) suggesting that interfacial adhesion weakened with increasing filler volume fraction. The loose particles in Figures 6(c) and 6(d) are consistent with interfacial debonding. Moreover, Figure 6(d) shows that BQW particles dispersed as aggregates at high filler content. This was anticipated for two reasons. First, inorganic particles tend to naturally cluster owing to the high polar surface energy. Second, since the FTIR results in Figure 2 showed that BQW particles are hydrophilic, and polymers are known to be hydrophobic, the agglomerated BQW particles were not sufficiently disintegrated during extrusion and therefore dispersed as aggregates [41].

### 3.4. Tensile Properties

#### 3.4.1. Tensile Strength

**Figure 9:**
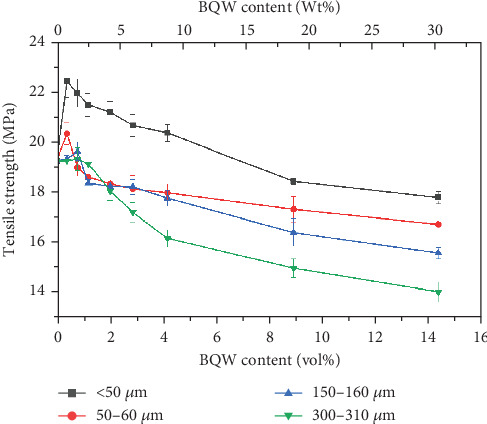
Tensile strength of recycled HDPE/BQW composites as a function of particle size and filler content.

Figure 9 illustrates the tensile behaviour of recycled HDPE/BQW composites as a function of particle size and filler content. Unfilled HDPE had a tensile strength of 19.270 ± 0.419 MPa. According to Figure 9, the addition of BQW initially increased the tensile strength of HDPE to a maximum for all particle sizes before strength began to deteriorate. Moreover, the maximum strength attained increased with decreasing particle size. For instance, composites with < 50-*μ*m particles recorded a 16.32% increase (from 19.270 ± 0.419 to 22.415 ± 0.639 MPa at 0.39 vol.% BQW content) while the corresponding increase for composites with 300–310-*μ*m particles was only 0.005% (from 19.270 ± 0.419 to 19.271 ± 0.436 at 0.39 vol.% BQW content). Even when tensile strength began to deteriorate at higher filler contents, composites with smaller-sized particles exhibited higher strength.

The initial increase and subsequent decline in tensile strength with increasing filler loading may be explained primarily in terms of the dispersion of the BQW particles within the HDPE matrix and the interfacial interaction between the two phases. At low filler loading, BQW particles were dispersed within the HDPE matrix to a good degree and fairly effective polymer–particle interfaces were formed. Thus, some of the stress borne by the HDPE matrix was distributed to the stronger BQW particles via these interfaces resulting in an overall improvement in strength. Although this good adhesion is not clear in Figure 7(a) (an SEM tensile fracture image of a composite with < 50-*μ*m particles at 0.39 vol.% BQW content) due to extensive polymer deformation and interfacial debonding, it can be observed in Figure 7(b) (an SEM image of the impact fracture surface of the same composite). At higher BQW concentrations, BQW particles dispersed as aggregates as shown in Figure 6(d) resulting in weak polymer–particle interfaces. Thus, little to no stress was transferred to the particles. On the contrary, these aggregates acted as voids and reduced the overall load-bearing area of the composite. Hence, stress was concentrated on the polymer matrix adjacent to these aggregates and the concentration was aggravated by the sharp edges of BQW particles. As a result, these composites failed at stresses below those of unreinforced HDPE.

The decline in strength at higher filler concentrations is consistent with what the authors [31] observed in PP/BQW composites and what de Moura et al. [29] reported for HDPE/GQW composites and attributed to poor filler distribution. It is noteworthy that there was no increase in strength in PP/BQW composites with < 50 and 300–310-*μ*m particles at lower filler loading in the previous study [31] as is the case in this study. Furthermore, while tensile strength increased by 5.51% for composites with 50–60-*μ*m particles at 0.79 vol.% BQW content in this study, the corresponding increase in PP/BQW composites was 2.74%. This indicates that HDPE formed stronger interfaces with BQW particles than PP. Additionally, de Moura et al. [29] did not observe any notable change in the strength of HDPE with GQW inclusions at low filler loading at any particle size despite reasonably good dispersion. This difference may imply that HDPE forms better interfaces with BQW than GQW.

Turcsanyi, Pukanszky, and Tudos [42] developed Equation (3) to estimate the variation of filler–polymer interactions with particle volume fraction.

(3) $$\begin{array}{c} \sigma_{yc}=\sigma_{ym}\frac{1-\varphi_{f}}{1+2.5\varphi_{f}}\exp\left(B\varphi_{f}\right) \end{array}$$

where *σ*_*ym*_ and *σ*_*yc*_ represent the tensile strength of the polymer matrix and the composite, respectively; *B* denotes the interfacial adhesion parameter between the filler and the matrix; and *φ*_*f*_  is the volume fraction of the filler. The value of *B* indicates the quality of the interfacial adhesion and depends on the interfacial bonding energy, particle density, and the surface area of the particles. In general, interfacial adhesion is poor when *B* is below one and strong when *B* is larger than 3. If a parameter *Q* is introduced to represent the relative tensile strength (*Q* = exp^*Bφ*_*f*_^), Equation (3) becomes

(4) $$\begin{array}{c} Q=\frac{\sigma_{yc}}{\sigma_{ym}}\left[\frac{1+2.5\varphi_{f}}{1-\varphi_{f}}\right] \end{array}$$

**Figure 10:**
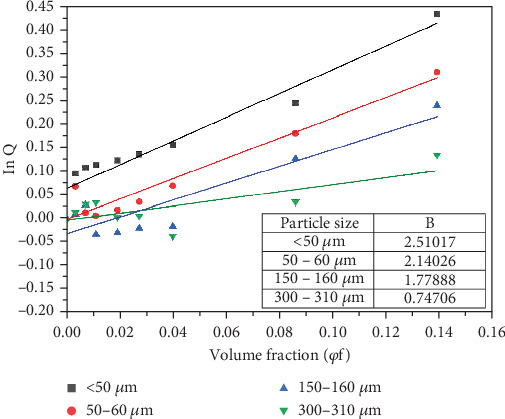
The relationship between the natural logarithm of the relative tensile strength *Q* and filler volume fraction.

The value of *B* can be calculated from the slope of the graph when the natural logarithm of parameter *Q* (ln *Q*) is plotted against the filler volume fraction (*φ*_*f*_) for each particle size as shown in Figure 10. The calculated values of the interfacial adhesion parameter in Figure 10 indicate that interfacial adhesion was poor for the 300–310-*μ*m particles but average for < 50-, 50–60-, and 150–100-*μ*m particles. Moreover, the value of *B* in Figure 10 increased with decreasing particle size. This observation is consistent with the tensile strength results in Figure 9 where on average, composites with smaller-sized particles exhibited relatively higher strength. Turcsanyi, Pukanszky, and Tudos' [42] model therefore has a generally good predictive value for the behaviour of these composites. The values of *B* obtained in this study are higher than those obtained in the earlier study [31]. This observation agrees with the earlier assertion that HDPE formed better interfaces with BQW particles than PP.

#### 3.4.2. Young's Modulus

**Figure 11:**
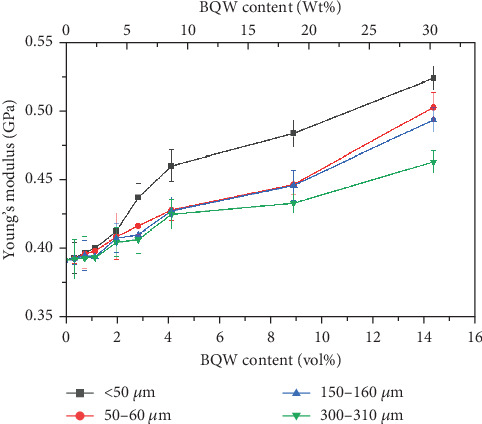
Young's modulus of recycled HDPE/BQW composites as a function of particle size and filler content.

Figure 11 shows that Young's modulus of recycled HDPE increased with increasing BQW content even at higher volume fractions. Since the quality of the interfacial bond also affects composite stiffness [43], this observation may appear to conflict with Figures 6(c) and 6(d) which showed poor interfacial adhesion between the two phases and agglomeration at higher filler contents. However, Mirjalili, Chuah, and Salahi [44] explained that particulate fillers restrain the molecular movements of polymers. This is reasonable since inorganic fillers have significantly higher stiffness than polymers and therefore do not stretch together with the polymer chains. Even the particle agglomerates observed at higher filler content likely acted as effective barriers to polymer chain motion.

In Figure 11, Young's modulus also improved with reducing particle size. In general, the difference in composite Young's moduli was not significant at very low filler contents and fell within the margins of error. However, it became consequential at higher volume fractions. For example, at 14.31 vol.% BQW content, Young's modulus of composites with < 50-*μ*m particles had improved by 33.4% whereas the corresponding increase for composites having 300–350-*μ*m particles was 18.1%. The general improvement in Young's modulus is lower in this study than in PP/BQW composites [31], and this may suggest that BQW particles were less effective barriers to HDPE than PP chain movements. It is worth noting that the increase in stiffness with reducing particle size was also reported by de Moura et al. [29] and Zhang et al. [45] in HDPE/GQW and HDPE/alumina composites, respectively, and is a consequence of the increased surface area for interfacial bonding when the particle size reduces.

#### 3.4.3. Elongation at Break

**Figure 12:**
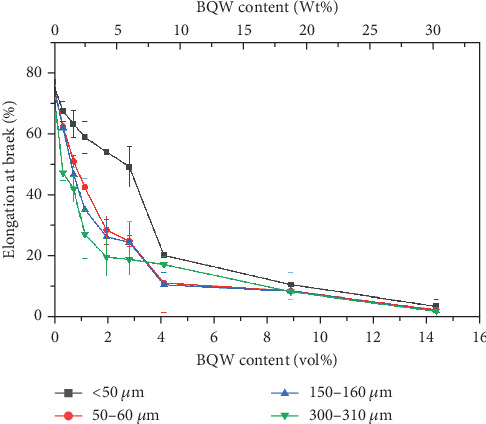
Elongation at break of HDPE/BQW composites as a function of particle size and filler content.

The decline in ductility with increasing BQW content in Figure 12 is consistent with what Khalaf [46] observed in virgin HDPE/calcium carbonate composites and reinforces the claim that rigid inorganic fillers impede the stretching and unwinding of polymer chains. However, de Moura et al. [29] observed the opposite effect in HDPE/GQW composites. This is likely because de Moura et al. [29] used GQW with significant amounts of biotite. Biotite is weak [47] and therefore provided little resistance to polymer chain movement. Three additional observations may be made in Figure 12. First, the drop in ductility was sharp at lower volume fractions but flattened out at higher volume fractions as it approached 0%. Second, the difference in ductility between the particle sizes is more distinct at lower filler contents but the curves almost coincide at higher filler content where particle agglomerates were observed. These two observations suggest a diminishing dependence of ductility on both filler volume fraction and particle size as filler content increases and was likely caused by particle agglomerates present in all particle sizes. Third, at lower filler contents where the difference in ductility between the particle sizes was distinct, composites with finer particles had slightly better ductility. This observation may imply that the smaller particles were less effective at blocking HDPE chain motion than larger particles.

### 3.5. Charpy's Impact Strength

**Figure 13:**
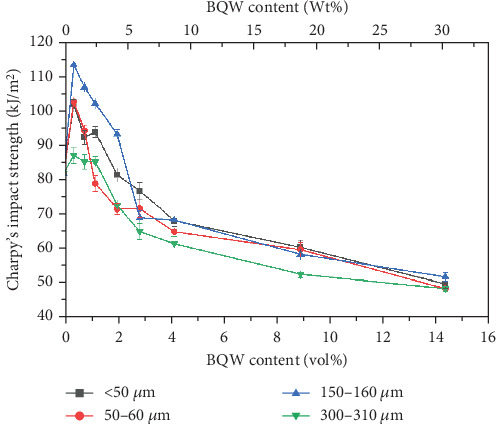
Charpy's impact strength of HDPE/BQW composites as a function of particle size and filler content.

Like tensile strength, Figure 13 shows that BQW particles improved the impact strength of HDPE at lower BQW content. Similarly, impact strength deteriorated at higher BQW loading. However, in this case, composites with 150–160-*μ*m particles rather than those with < 50-*μ*m particles registered the highest increase in impact strength. Additionally, while the difference in tensile strength between the different particle sizes was distinct at higher filler loading, the corresponding difference in impact strength is not clearly defined. Unfilled HDPE had a higher impact strength of 81.667 ± 0.4327 kJ/m^2^ compared to the 53.391 ± 1.227 kJ/m^2^ exhibited by the PP used in the previous study [31] despite PP having a higher tensile strength. This is likely due to the high plastic deformation observed in the impact fracture surface of HDPE in Figures 8(a) and 8(b) which demonstrates that considerable impact energy was required to deform HDPE before impact cracks could propagate through the polymer. Deformation was less pronounced in PP. Figures 8(c) and 8(d) may provide important insights into why BQW particles enhanced the impact strength of HDPE at low filler contents. First, the protruding particle in Figure 8(c) indicates that BQW particles firmly attached to the HDPE matrix acted as barriers to impact crack propagation. Thus, extra energy was required to circumvent these particles than if they were not present. Second, the cavity in Figure 8(d), likely left by a dislodged particle, suggests that some impact energy was used to dislodge BQW particles. However, at higher BQW concentrations, the particle aggregates observed in Figure 6(d) accelerated crack initiation and propagation such that these composites failed under less impact energy than unfilled HDPE. Nonetheless, the material retained desirable toughness given the ductile failure observed in Figures 6(c) and 6(d). This overall reduction in impact strength at higher filler concentrations was also observed by Shagwira et al. [28] in HDPE/GQW composites.

### 3.6. Compressive Properties

#### 3.6.1. Compressive Strength

HDPE has a low compressive strength and is, therefore, not commonly used under compression. In most studies found in literature, recycled HDPE was tested under compression only when it was incorporated as a minor phase in concrete [48–51]. These studies showed that the compressive strength of concrete generally decreased with increasing HDPE content as was expected. However, since some of the targeted applications of the composite material under study are paver blocks which are subjected to compressive loading in service, it was worthwhile to investigate how the addition of BQW would alter the compressive strength of recycled HDPE.

**Figure 14:**
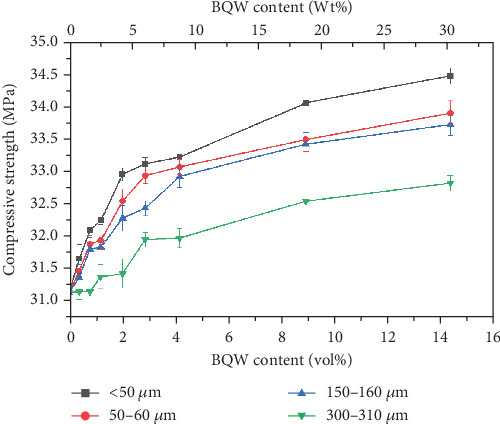
Compressive strength of HDPE/BQW composites as a function of particle size and filler content.

Figure 14 shows that adding BQW to HDPE was beneficial since compressive strength increased with filler content for all composites. These results indicate that BQW particles fortified HDPE by bearing some compressive load. The smallest particle size registered the largest enhancement in strength (10.6% increase in strength at 14.31 vol.% BQW content) pointing to the significance of the interfacial contact area in compressive stress transfer. In comparison, the corresponding increase for composites with the largest particles was 5.4%. Similar results were observed by the authors [31] in recycled PP/BQW composites.

#### 3.6.2. Compressive Modulus

**Figure 15:**
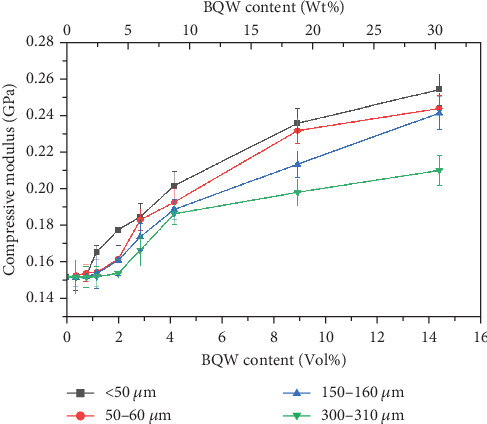
Compressive modulus of HDPE/BQW composites as a function of particle size and filler content.

The variation of the compressive modulus of HDPE with particle size and volume fraction in Figure 15 mirrors Young's modulus results in Figure 11, illustrating that BQW particles and particle aggregates restrained HDPE chain movements in both tension and compression. This effect increased with decreasing particle size in both cases. However, the results also demonstrate that BQW particles were far more effective at enhancing the compressive modulus of HDPE than its Young's modulus. For instance, the compressive modulus of HDPE increased by 66.6% for the < 50-*μ*m particles at 14.31 vol.% BQW content while the corresponding increase in Young's modulus was 33.4%.

### 3.7. Hardness

**Figure 16:**
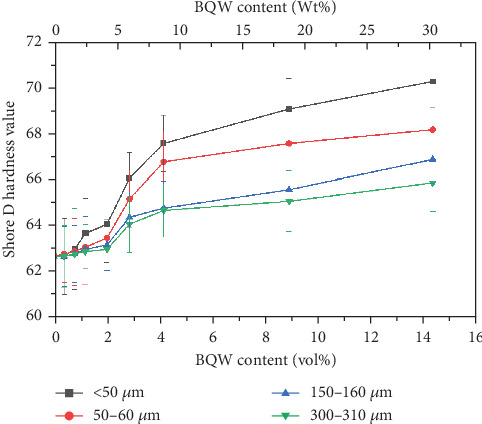
Shore *D* hardness values of HDPE/BQW composites as a function of particle size and filler content.

Table 1 showed that the primary constituent of BQW is silica which is a hard ceramic material. It was therefore anticipated that BQW would enhance the hardness of HDPE and this is evident in Figure 16 and agrees with Shagwira's et al. [28] results for HDPE/GQW composites. For the smallest particle size, Shore *D* hardness increased by 12.1% at 14.31 vol.% BQW content. However, it was 5.1% for the largest particle size at the same filler content. This difference may be explained by the fact that there was a better distribution of the finer particles in the matrix and therefore more particles per unit volume of the composite. Nonetheless, the large and overlapping error bars for all particle sizes signify that the particles were not sufficiently distributed to impart uniform hardness.

### 3.8. Water Absorption Properties

Dhakal, Zhang, and Richardson [52] demonstrated that significant water diffusion in hemp fibre–reinforced polyester composites degraded their interfacial bond and deteriorated mechanical properties. Guo, Finkenstadt, and Nimmagadda [53] observed similar results in HDPE/carbon fibre composites. In general, three mechanisms are responsible for water diffusion in particulate polymer composites. First, water can diffuse into the microgaps between polymer chains. Second, water can diffuse via capillary action into voids between the particle–polymer interfaces or voids between particles in the case of agglomerates. Third, water can diffuse into microcracks that form on the polymer during composite preparation or microcracks or pores in the filler particles [52].

**Figure 17:**
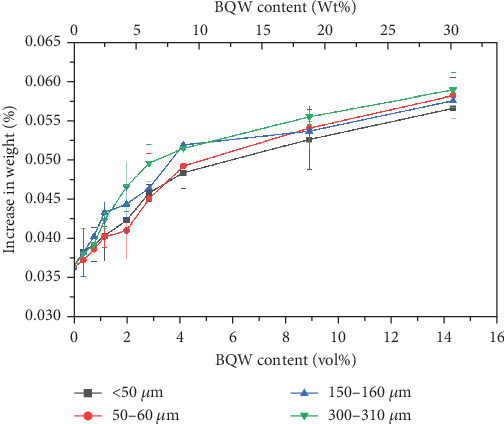
The percentage increase in the water absorbed by HDPE/BQW composites as a function of particle size and filler content.

Figure 17 shows that the water uptake for unfilled HDPE was very low at 0.0363% ± 0.0024% and this may suggest that some water molecules diffused between HDPE chains. There is an increase in water absorption with filler content for all particle sizes, and this is generally consistent with Shagwira et al.'s [28] results. However, this increase is not consequential and may imply that water only diffused into microcracks and pores on surface particles and did not diffuse into the composite. If this was the case, it is unlikely that water absorption would have any significant effect on the mechanical properties of these composites. It is also notable that the relationship between water absorption and particle size is less defined than it was for mechanical properties.

### 3.9. Density

**Figure 18:**
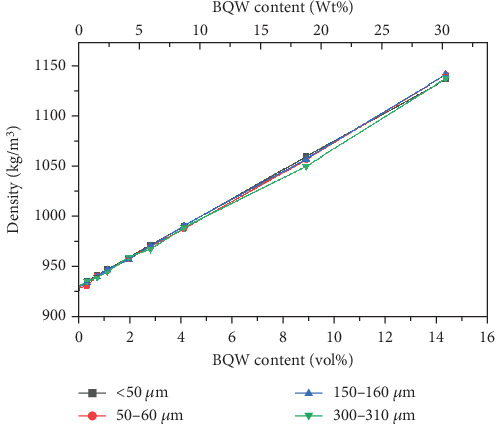
Density of HDPE/BQW composites as a function of particle size and filler content.

Recycled HDPE and BQW exhibited densities of 930.1 ± 0.1182 kg/m^3^ and 2387 ± 1.3321 kg/m^3^, respectively.  Figure 18 illustrates that the addition of BQW increased the density of HDPE linearly in a manner consistent with the rule of mixtures. Moreover, the increase was significant (by 22.65 at 14.31 vol.% BQW content) owing to the relatively higher density of BQW. The slight differences in densities between the particle sizes may have been caused by errors in reading the volume of water during experimentation or minor voids may have been present within the composites.

## 4. Conclusions

In this study, the feasibility of recycling HDPE and BQW into a material that may be used to make alternative construction materials such as roofing tiles and paver blocks was investigated. No new chemical bonds were detected by FTIR between HDPE and BQW hence the bonding between them was primarily mechanical. However, the TGA results demonstrated that incorporating BQW generally enhanced the thermal stability of recycled HDPE. The SEM images revealed poor bonding between HDPE and BQW particles. This negatively impacted the tensile and impact strengths of the composites, especially at higher filler contents where particle agglomerates were observed. Nonetheless, the material still retained desirable toughness. Further study may be necessary to find an appropriate compatibilizing agent to enhance adhesion between the two materials. The stiffness, compressive strength, compressive modulus, hardness, and density of HDPE improved significantly with increasing BQW content, and further improvements can be expected at higher filler loading. Although incorporating BQW in recycled HDPE increased its water absorption, the increase was not significant and therefore, the material is suitable for use in outdoor settings. Specifically, the water absorption of the material meets the recommended standard for concrete roofing tiles and paving blocks set by ASTM C1492 and ASTM C1319, respectively. Moreover, the minimum compressive strength recommended by ASTM C1319 of 35 MPa could be achieved at a higher BQW content (since the material had a compressive strength of 34.5 MPa at 14.31 vol.% BQW content). This study therefore demonstrates that BQW is a suitable filler for recycled HDPE for producing sustainable construction materials. However, further testing such as weathering and abrasion resistance may be necessary to assess the durability of the material.

## Figures and Tables

**Table 1:** XRF analysis of the BQW [31].

**Compound present**	**A** **l**_2_ **O**_3_	**C** **a** **O**	**F** **e** **O**	**K**_2_ **O**	**M** **g** **O**	**M** **n** **O**	**P**_2_ **O**_5_	**S** **i** **O**_2_	**T** **i** **O**_2_	**Z** **r** **O**_2_	**LOI**
Quantity (wt%)	17.9	1.87	4.36	6.97	1.28	0.28	0.19	65.9	0.70	0.11	0.44

**Table 2:** Formulations used to prepare recycled HDPE/BQW composites.

**Formulation**	**Recycled HDPE**		**Basaltic quarry waste (BQW)**	
	**Wt%**	**Vol.%**	**Wt%**	**Vol.%**
1	100	100	0	0
2	99	99.61	1	0.39
3	98	99.21	2	0.79
4	97	98.81	3	1.19
5	95	98.00	5	2.00
6	93	97.15	7	2.85
7	90	95.85	10	4.15
8	80	91.12	20	8.88
9	70	85.69	30	14.31

*Note:* The densities of HDPE and BQW used in calculating the volume fractions were 930.1 kg/m^3^ and 2387 kg/m^3^ respectively.

**Table 3:** Summary of functional groups detected in BQW.

**Band (cm** ^**−1**^ **)**	**Bond (vibration mode)**	**Compound**
3935	M-OH (stretching vibrations)	Biotite group
3863	M-OH (stretching vibrations)	Biotite group
3800	M-OH (stretching vibrations)	Biotite group
3731	M-OH (stretching vibrations)	Biotite group
3600–3200	H-O-H (stretching vibration)	Water
2100–2350	C*Ξ*C (stretching vibrations)	Organic compound
1644	(CO_3_)^−2^ (asymmetrical stretching)	Calcite
1556	(CO_3_) ^−2^ (asymmetrical stretching)	Calcite
1414	(CO_3_) ^−2^ (asymmetrical stretching)	Calcite
1021	Si-O-Si (symmetrical stretching)	Quartz
746	Si-O-Si (symmetrical stretching)	Quartz
482	Si-O-Si (asymmetrical bending)	Quartz

**Table 4:** Thermogravimetric data for the investigated samples.

**Sample**	**BQW (vol.%)**	**T**_**o**_ ** (°C)**	**T**_1_ ** (°C)**	**T**_2_ ** (°C)**	**T**_3_ ** (°C)**	**Residue at 600 °C (wt%)**
1	0	246	292	415	432	24.48
7	4.15	224	290	420	448	28.40
8	8.88	234	262	431	468	39.87
9	14.31	238	275	416	493	37.42

*Note:T*_o_ = mass loss onset temperature; *T*_1_ = peak temperature of the first stage of decomposition; *T*_2_ = peak temperature of the second stage of decomposition; *T*_3_ = peak temperature of the third stage of degradation.

## Data Availability

The data used to support the conclusions of this study are included in this paper.

## Nomenclatures

QW: quarry waste
GQW: granitic quarry waste
BQW: basaltic quarry waste
*σ*_*ym*_: tensile strength of the polymer matrix
*σ*_*yc*_: tensile strength of the composite
*φ*_*f*_: filler volume fraction
*B*: interfacial adhesion parameter
*E*_*c*_: Young's modulus of the composite
*E*_*m*_: Young's modulus of the matrix
*E*_*p*_: Young's modulus of the particles
*V*_*m*_: volume fraction of the matrix
*V*_*p*_: volume fraction of the particles
